# A decision-analytic approach to define poor prognosis patients: a case study for non-seminomatous germ cell cancer patients

**DOI:** 10.1186/1472-6947-8-1

**Published:** 2008-01-03

**Authors:** Merel R van Dijk, Ewout W Steyerberg, J Dik F Habbema

**Affiliations:** 1 Department of Public Health, Erasmus MC, PO Box 2040, 3000 CA Rotterdam, The Netherlands

## Abstract

**Background:**

Classification systems may be useful to direct more aggressive treatment to cancer patients with a relatively poor prognosis. The definition of 'poor prognosis' often lacks a formal basis. We propose a decision analytic approach to weigh benefits and harms explicitly to define the treatment threshold for more aggressive treatment. This approach is illustrated by a case study in advanced testicular cancer, where patients with a high risk of mortality under standard treatment may be eligible for high-dose chemotherapy with stem cell support, which is currently defined by the IGCC classification.

**Methods:**

We used published literature to estimate the benefit and harm of high-dose chemotherapy (HD-CT) versus standard-dose chemotherapy (SD-CT) for patients with advanced non-seminomatous germ cell cancer. Benefit and harm were defined as the reduction and increase in absolute risk of mortality due to HD-CT respectively. Harm included early and late treatment related death, and treatment related morbidity (weighted by 'utility').

**Results:**

We considered a conservative and an optimistic benefit of 30 and 40% risk reduction respectively. We estimated the excess treatment related mortality at 2%. When treatment related morbidity was taken into account, the harm of HD-CT increased to 5%. With a relative benefit of 30% and harm of 2 or 5%, HD-CT might be beneficial for patients with over 7 or 17% risk of cancer specific mortality with SD chemotherapy, while with a relative benefit of 40% HD-CT was beneficial over 5 and 12.5% risk respectively. Compared to the IGCC classification 14% of the patients would receive more aggressive treatment, and 2% less intensive treatment.

**Conclusion:**

Benefit and harm can be used to define 'poor prognosis' explicitly for non-seminomatous germ cell cancer patients who are considered for high-dose chemotherapy. This approach can readily be adapted to new results and extended to other cancers to define candidates for more aggressive treatments.

## Background

The prognosis of a cancer patient is of key importance in the choice of more or less aggressive treatment. Prognostic estimates can be based on extent of disease, as for example reflected in TNM stage, on age and comorbidity, and on specific characteristics, such as values of tumour markers [1]. Prognostic classifications can facilitate decision-making by grouping patients with a similar prognosis. Poor prognosis patients may be considered candidates for more aggressive treatment strategies, while good prognosis patients may be treated with less burdensome interventions, for example by less toxic chemotherapy regimens [2,3]. Prognostic classifications use estimated survival to identify poor prognosis patients eligible for alternative treatments. However this approach only implicitly takes the possible side effects of an alternative treatment into account. Ideally both the expected gain in survival (benefit) and the toxic side effects or burden due to treatment (harm) are considered [4].

We apply a decision analytic approach proposed by Glasziou and Irwig (1995) in which both benefit and harm of an alternative treatment are explicitly specified and weighed to determine which patients could profit from this alternative treatment strategy [4].

**Figure 1:**
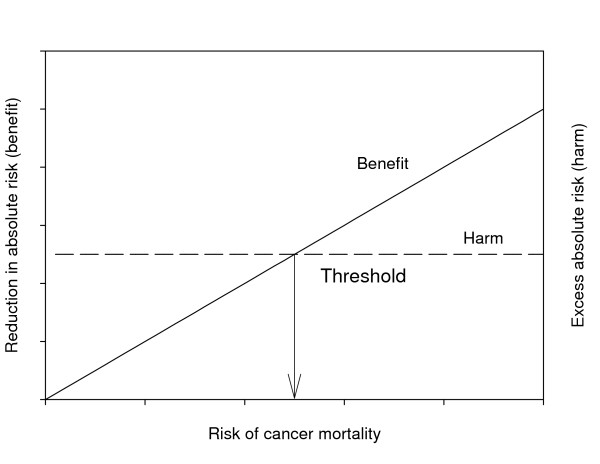
Benefit and harm of treatment, expressed on the same scale. Benefit of treatment (reduction in absolute risk) increases with risk, while harm of treatment (excess absolute risk, e.g. due to toxicity of treatment) is constant. Net benefit occurs only when risk is above the threshold. Adapted from Glasziou & Irwig (1995).

The decision analytic approach is illustrated in Figure 1. Benefit of treatment is the reduction in absolute risk of cancer mortality due to treatment. Benefit increases linearly with risk of cancer mortality assuming that patients with the highest risk have most to gain. Harm is the increase in absolute risk of treatment mortality (e.g. related to toxicity) due to treatment. The level of harm is the same for all patients, assuming that for example the toxicity of treatment is independent of prognosis. Patients are candidates for more aggressive treatment when their risk of cancer mortality is above the threshold, i.e. when benefit is higher than harm [4].

As an example we consider high-dose chemotherapy (HD-CT) as first line treatment to improve survival of patients with nonseminomatous germ cell cancer. Several non-randomised trials reported a higher survival for poor prognosis patients treated with HD-CT as first line treatment (including etoposide, ifosfamide, cisplatin) with autologous stem cell support, compared to standard-dose chemotherapy (SD-CT) (including bleomycin, etoposide, cisplatin) [5-7]. Furthermore, HD-CT is currently considered in two RCTs by the European Organisation for Research and Treatment of Cancer (EORTC) and by the US intergroup [8,9]. However, HD-CT is related to a higher toxicity, both during treatment (e.g. granulocytopenia, anaemia, nausea/vomiting, diarrhoea), shortly after treatment (e.g. pulmonary toxicity) and long after treatment (e.g. leukaemia, cardiovascular disease) [5,10].

So far studies on HD-CT focus on patients with a poor prognosis according to the International Germ Cell Consensus (IGCC) Classification [11]. The IGCC classification combined 5 risk factors to define a good, intermediate and poor prognosis group based on survival. Good prognosis patients are considered eligible for less intensive treatment reduce treatment related toxicity [12], intermediate prognosis patients usually receive standard treatment, and poor prognosis patients are considered candidates for more aggressive treatment. However, as many other prognostic classifications, the IGCC classification only considers survival in determining different prognosis groups and does not take the possible (long-term) harm of alternative treatments such as high-dose chemotherapy into account. By taking both expected harm and benefit into account we can more precisely determine which subgroup of patients might profit from high-dose chemotherapy.

The aim of this study is to use a decision-analytic approach to determine how high the risk of patients with nonseminomatous germ cell cancer should be in order to profit from high-dose chemotherapy with stem cell support. Estimates of benefit and harm of high-dose chemotherapy were based on currently available literature.

## Methods

Of the different high-dose chemotherapy (HD-CT) treatment strategies currently investigated we considered the benefit and harm of the HD-CT approach by the German testicular cancer group [5].

We considered benefit and harm till 10 years after treatment, since longer-term evidence is scarce.

### Benefit

Benefit is based on the reduction in relative risk due to HD-CT compared to standard chemotherapy.

Benefit is expressed as:

1 - (R_C-MORT HD-CT_/R_C-MORT SD-CT_)

where R_C-MORTHD-CT _is the risk of cancer mortality with HD-CT and R_C-MORTSD-CT _the risk of cancer mortality with standard chemotherapy. This relative risk reduction translates into a decrease in absolute risk of cancer mortality at the patient level. When HD-CT results in a relative risk reduction of 25%, absolute risk decreases 10% for patients with a risk of cancer mortality of 40% (0.25 × 40), whereas for a patient with a risk of cancer mortality of 80% the absolute risk reduction is 20% (0.25 × 80).

Although benefit should preferably be based on results of RCTs it will take several more years before the results of RCTs comparing HD-CT to SD-CT become available. To estimate risk of cancer mortality due to HD-CT and SD-CT we therefore selected three observational studies; two reporting on patients recently treated with SD-CT and one study describing the long-term results of the HD-CT approach by the German testicular cancer group [5,13,14]. The selection of these studies was based on an extensive search of the literature as in a previously published meta-analysis [15].

These observational studies reported on either 5-year or 10-year survival. To estimate benefit we need the risk of cancer mortality due to SD-CT and HC-CT 10 years after treatment.

We therefore translated survival into risk of cancer mortality at 10 years.

Firstly, overall survival (S_OVERALL_) in each study was translated to risk of overall mortality due to treatment (R_OVERALL_).

R_OVERALL _= 1 - S_OVERALL_

From the overall risk of mortality we determine the risk of cancer mortality (R_C-MORT_) by subtracting risk of treatment mortality (R_T-MORT_). We ignore mortality due to other causes since testicular cancer patients are relatively young.

R_C-MORT _= R_OVERALL _- R_T-MORT_

Finally, we assumed that the relative increase in risk between 5 years and 10 years after treatment was 20% and increased the risk of cancer mortality accordingly [11]. The resulting estimates of cancer mortality 10 years after treatment of the two studies on SD-CT were combined in a weighted average by study size.

### Harm

Harm is the excess risk of mortality due to HD-CT and is assumed to remain comparatively constant. We considered the excess risk of mortality and morbidity using published literature.

Treatment mortality consisted of early treatment mortality (<6 months) and late treatment mortality (>6 months). We based late treatment mortality (R_LATE T-MORT_) on the incidence of long-term complications and fatality of these long term complications once they occur. Fatality was assumed to be identical for patients treated with HD-CT or SD-CT once a complication occurred, although no information was available on similarity of fatality between patients treated with either SD-CT or HD-CT.

The excess risk of late treatment mortality is the difference in incidence multiplied by the estimated fatality:

ΔR_LATE T-MORT _= (incidence_HD-CT _- incidence_SD-CT_) × fatality.

Late treatment morbidity (R_LATE T-MORB_) was made comparable to mortality by weighing complications by their utility value. Utility (U) is a measure of health related quality of life, ranging from 0 to 1, where a weight of 1 corresponds to perfect health and a weight of 0 corresponds to a health state judged equivalent to death [16]. By expressing long-term complications in utilities, treatment related morbidity could be directly compared with treatment related mortality.

We estimated late treatment morbidity for SD-CT and HD-CT by combining the incidence and utilities of long-term complications up to 10 years after treatment. We obtained utilities for long term complications from available literature [16].

The risk of excess late treatment morbidity for surviving patients is:

ΔR_LATE T-MORB _= (incidence_HD-CT _- incidence_SD-CT_) × (1-U) × (1-fatality).

### Sensitivity analysis

We considered a conservative and an optimistic scenario for benefit, since only observational data were available. Further, a constant relative risk reduction assumes a linear relationship between benefit and risk, where benefit is absent for patients with no risk, and maximal for patients with 100% risk of cancer mortality. Since treatment effect is not necessarily similar for patients at varying risks we also considered a non-linear relationship between benefit and risk, in which benefit is absent for patients with no risk or 100% risk and maximal for patients with a 50% risk of cancer mortality. We determined the threshold for such a parabolic relation between benefit and risk, for both the optimistic and conservative scenario. Finally, we calculated treatment thresholds for more aggressive therapy when benefit and harm were varied over wide ranges. All analyses were performed in Microsoft Excel 2000.

## Results

### Benefit

The three observational studies on which our estimate of benefit of HD-CT was based are presented in Table 1. Sonneveld et al. reported 10-year disease specific survival of 66% for 22 patients treated with SD-CT in their hospital between 1987 and 1996 [17]. A RCT comparing standard dose bleomycin-etoposide-cisplatin (BEP) with standard dose etoposide-ifosfamide-cisplatin (VIP) reported a 5-year overall survival of 60% for 181 poor prognosis patients [13]. Schmoll et al. reported five-year survival of 73% for 182 patients treated with HD-CT between 1993 and 1999 [5].

**Table 1:** Survival and early treatment related death in non-seminomatous germ cell cancer patients treated with high-dose (HD) or standard-dose (SD) chemotherapy

Reference	Treatment	Year treatment	N	S_OVERALL_	F-up	R_OV-MORT_	Early toxic death^2^	R_C-MORT_	R_C-MORT 10 yrs_
Hinton *et al.* [13]	SD	1987–1992	181	60%	5	40%	3%	37%	44%
Sonneveld *et al.* [17]	SD	1987–1996	22	66%^1^	10	34%	NA	31%	31%
Schmoll *et al.* [5]	HD	1993–1999	182	73%^1^	5	27%	4%	23%	28%

S_OVERALL _= Overall survival at year of follow-up

F-up = follow-up in years

R_OV-MORT _= Risk of overall mortality at year of follow-up

R_C-MORT _= Risk of cancer mortality at year of follow-up

R_C-MORT 10 yrs _= Risk of cancer mortality 10 year after treatment

1 disease specific survival

2 early toxic death [5]: neutropenic infections (decreased white blood cells) and septic multi-organ failure. HD toxic death: any death occurring within 100 days from grafting and not directly related to the disease itself.

### Harm

Early treatment related mortality was 3% for patients treated with SD-CT in RCT [18]. This is concordant with an early treatment related mortality of 3% reported in other series [19,20]. HD-CT early treatment related mortality was 4%. The European Group for Blood and Marrow Transplantation (EBMT) Solid Tumours registry has recently reported an update of the mortality rate of germ cell tumour patients treated in Europe between 1990 and 1999. The rate of toxic death, defined as any death occurring within 100 days from grafting and not related to the disease itself, declined from 8% in 1990 to 3% in 1999 (overall 5%) [20]. We estimate the excess early treatment mortality as 1% (4-3%).

Table 2 lists the most common complications due to treatment of non-seminomatous germ cell cancer [10,21]. For each complication the incidence for SD-CT and HD-CT is given and the suspected agent. Leukaemia is the main cause of late treatment mortality in patients treated for NSGCT. More patients are expected to develop leukaemia after HD-CT than SD-CT (1.5 vs. 0.5%). With a mortality of 70% for leukaemia, this results in a difference in late treatment mortality of 0.7% [10,22,23]. Cardiovascular disease further contributes to treatment mortality of patients treated for NSGCT [10,24,25]. The incidence of cardiovascular disease is estimated as 7% for SD-CT patients. We estimated the incidence of cardiovascular disease at, 10% for HD-CT patients, although no firm empirical estimates were available for HD-CT. With a fatality of 10% this results in 0.3% excess mortality. The combination of early and late treatment related mortality resulted in an estimated harm of 2%.

**Table 2:** Incidence, mortality and utility of long term complications due to high-dose (HD) or standard-dose (SD) chemotherapy for non-seminomatous germ cell cancer.

Morbidity	References	Incidence		Suspected agent	Mortality	Change in mortality^3^	Utility^4^	Change in morbidity^5^
		SD	HD					

Therapy related leukaemia	[10, 22, 23]	0.5%	1.5%	Etoposide (< 2 g/m^2^, > 2 g/m^2^)	70%	0.7%	0.90	0.03%
Vascular toxicity	[10, 16, 24, 25]							
Raynaud's phenomenon		25%	>25%	Bleomycin			-	-
Cardiovascular disease		7%	10%	Cisplatin	10%	0.3%	0.7	0.81%
Neurotoxicity	[5, 10, 21]							
Peripheral neuropathy		4%	5%	Cisplatin			-	-
Ototoxicity		5%	65%	Cisplatin (<400 mg/m^2^, > 400 mg/m^2^)			-	-
Nephrotoxicity	[5, 10, 16]							
Renal failure		1%	4%	Cisplatin			0.6	1.2%
Hypertension		10%	24%	Cisplatin (<400 mg/m^2^, > 400 mg/m^2^)			0.99	0.14%
Gonadal toxicity	[10, 16, 26, 41]							
Infertility^1^		50%	>50%	Cisplatin				
Sexual functioning^2^		15%	27%				0.92	0.96%
Total						**1%**		**3.14%**

^1 ^oligospermia/azoospermia

^2 ^sexual dissatisfaction

^3 ^Change in mortality calculated as (incidence_HD-CT _- incidence_SD-CT_) × fatality

^4 ^Utility ranges from 0–1 and is a measure of health related quality of life

^5 ^Change in morbidity calculated as (incidence_HD-CT _- incidence_SD-CT_) × (1-U) × (1-fatality).

Other long-term complications vary from relatively mild (Raynaud's phenomenon, ototoxicity) to severe (renal failure) [5,10,26]. In estimating the difference in long-term morbidity between SD-CT and HD-CT we only took the more severe complications into account. No utility was known for acute myeloid leukaemia. Although physical and emotional functioning of long term leukaemia survivors is near normal, sexual functioning and fertility is often affected [27]. We therefore estimated a utility of 0.9 for treatment related leukaemia.

The overall difference in utility weighted long-term morbidity was 3.1%. The total harm due to HD-CT was approximately 5% (excess mortality 2% + excess morbidity 3.1%).

### Treatment thresholds for HD-CT

To determine the benefift of high-dose over standard dose chemotherapy 10 years after treatment we had to determine the risk of cancer mortality for both treatment strategies.

Firstly, we determined risk of overall mortality (see formula 2) for the three observational studies, which were 40, 34 and 27% respectively (Table 2) [5,13,17]. By subtracting treatment related mortality (formula 3), 4% for high-dose chemotherapy and 3% for standard chemoterhapy, we obtained the risk of cancer mortality (37, 31 and 23% respectively). To obtain the risk of cancer mortality 10 years after treatment the estimates from Hinton et al. and Schmoll et al. were increased with 20%.

Combined, the 203 patients treated with SD-CT had an estimated 10-year risk of cancer mortality of 43%, which was substantially higher than that for the 182 patients treated with HD-CT chemotherapy (10-year risk of cancer mortality 28%). The pooled estimate of benefit (see formula 1) is 35% (RRR = 1 - (28%/43%)). For our conservative scenario we assume a benefit of 30% and for our optimistic scenario a benefit of 40%.

**Figure 2:**
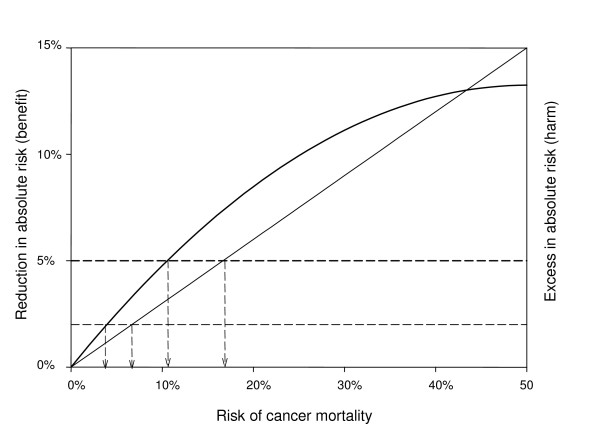
Linear benefit (30%) and non-linear benefit (30%) vs. harm of high dose chemotherapy, with harm defined as 10-year treatment related mortality (2%) or mortality plus morbidity (5%). The arrows indicate the thresholds to define poor prognosis (7% and 17% respectively for linear benefit, 4% and 11% respectively for non-linear benefit).

At a benefit of 30% and only treatment related mortality included in our estimate of harm (2%), patients with only 7% risk of cancer mortality or higher should be treated with HD-CT (Figure 2). With a benefit of 40% the treatment threshold was as low as 5%.

When we also take treatment related morbidity into account in our estimate of harm (5%) and benefit is 30%, patients with a 17% risk of cancer mortality or higher should be treated with HD-CT (Figure 2). With a benefit of 40% the treatment threshold was 12.5%.

When we assumed a non-linear benefit of 30% and a harm of either 2 or 5% treatment thresholds were 4 and 11% respectively (Figure 2). With a non-linear benefit of 40% threshold values were below 10% (3% and 8% respectively).

**Figure 3:**
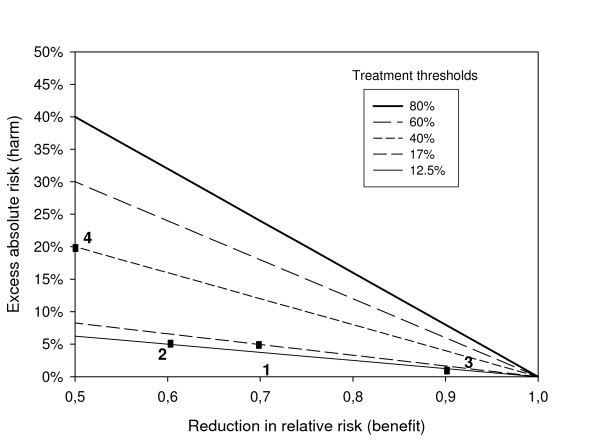
Thresholds according to risk with standard treatment for a range of hypothetical benefits (reduction in relative risk, RR) and harms associated with a more aggressive treatment. 1. benefit 30%, harm = 5%, threshold = 17% (- - -), 2. benefit 40%, harm = 5%, threshold = 12.5% (--), 3. benefit 10%, harm = 1%, threshold = 12.5% (--), 4. benefit 50%, harm = 20%, threshold = 40% (----).

The estimates of benefit and harm determine the treatment thresholds as shown in Figure 3 for treatment benefits from 0 to 50% and harms from 0% to 40%. For non-seminomatous germ cell cancer patients an estimated benefit of 30% and harm of 5% resulted in a threshold of 17% (block 1). When we assumed a benefit of 40%, with the same harm of 5%, the threshold decreased to 12.5% (block 2). The same threshold could be obtained with a smaller benefit, and a much smaller harm, for example 10% and 1% (block 3). We could also consider more harmful therapies, which would naturally only be considered for types of cancer with a very poor prognosis. With harm as high as 20% and a benefit of 50%, the treatment threshold for such patients is a 40% risk of cancer mortality (block 4).

## Discussion

We illustrated how decision analysis can explicitly assist in defining poor prognosis testicular cancer patients who have a net benefit of high-dose chemotherapy (HD-CT) with stem cell support. Based on the currently available literature we considered a conservative estimate of 30% for the benefit and an optimistic estimate of 40%. We estimated a harm of 5%, based on both treatment mortality (2%) and treatment morbidity expressed in utilities (3%). Even with a conservative estimate of 30% for the benefit of treatment, and taking both treatment related mortality and morbidity into account, patients with a risk of cancer mortality of 17% or higher might already benefit from HD-CT. With a benefit of 40% this threshold was reduced to 12.5%. When we assumed benefit to be nonlinear, treatment thresholds were 11 and 8% for benefit of 30 and 40% respectively. Although this decision analysis was specific for the defining high-risk patients with germ cell tumors, it is in line with the more general approach for the selection of patients for clinical trials described by Vickers *et al.*[28].

To what extent is the group of patients above the threshold comparable to the poor prognosis patients as defined by the IGCC classification?

The IGCC classification does not explicitly use risk thresholds to determine prognosis groups.

However, we can use previously developed multivariable models to study the risk distribution within the 3 IGCC classification groups [29,30].

This also allows us to determine how many patients in each prognosis group have a risk above the treshold of 17% and how many patients have a risk below the treshold, i.e. for which patients does the use of the treatment treshold based on our decision analysis result in a change in treatment.

**Figure 4:**
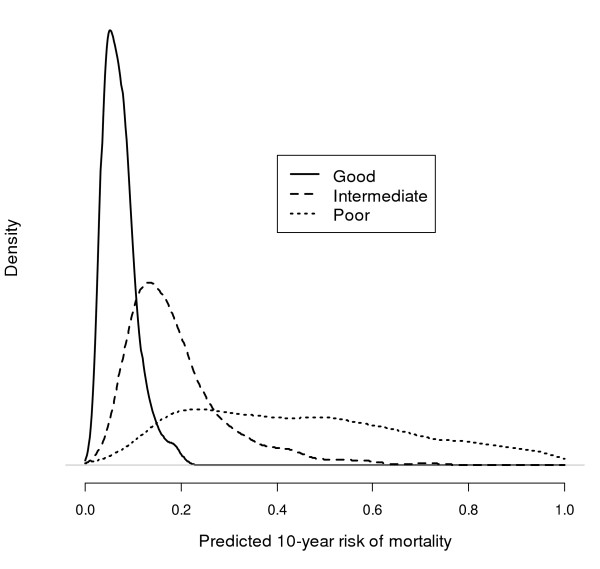
Distribution of predicted 10-year risk of mortality for the good, intermediate and poor prognosis groups of the IGCC classification. Mean risk of good, intermediate and poor prognosis were 7, 19 and 46% respectively.

The mean 10-year predicted risks of mortality of the good, intermediate and poor prognosis groups were 7, 19 and 46% respectively (Figure 4). We can define a threshold for the good prognosis patients such that the number of patients is identical to the number with the IGCC classification, and similarly for the poor prognosis group. The risk thresholds were 11% and 28% between the good and intermediate prognosis group, and between the intermediate and the poor prognosis group respectively.

According to the threshold, 881 of 3048 patients (29%) should be treated with HD-CT. Compared to the IGCC classification 28 good prognosis patients (1%) and 409 intermediate prognosis patients (13%), who would get SD-CT according to the IGCC classification, have a risk above the 17% threshold and therefore should get HD-CT. Fifty-one poor prognosis patients, who would get HD-CT according to the IGCC classification, have a risk below the threshold and should therefore get SD-CT.

The IGCC classification and our decision analysis hence largely disagree on intermediate prognosis patients as candidates for HD-CT. In the future, a more refined prognostic classification is desirable, with prognostic groups defined in more detail and with more powerful predictors, e.g. new biomarkers [31,32].

Although we considered a conservative and optimistic estimate of the benefit of HD-CT our estimate may still be too optimistic. Differences in treatment other than HD-CT may have affected the difference in survival between patients treated with SD-CT and patients treated with HD-CT. Firstly the patients treated with SD-CT were mainly treated in the US whereas patients treated with HD-CT were treated in Germany. However, the estimated risk of cancer mortality for SD-CT is in line with the IGCC survival estimate for poor prognosis patients adjusted for year of treatment, which is based on patients treated in both Europe and the US [15]. Secondly, patients treated with SD-CT were treated earlier than patients treated with HD-CT. Improvements over time in second line treatment may have effected the difference in survival [18].

Our estimate of harm may be too low. We estimated harm due to treatment related mortality and morbidity at 10 years after treatment. Direct estimates of early treatment mortality were available for both SD-CT and HD-CT. However information on long term complications is merely available for SD-CT, and limited for HD-CT. As a consequence our estimate of etoposide induced leukaemia, which is very difficult to cure, may be too low.

Similarly, the harm due to complications such as cardiovascular disease and hypertension may be higher since they pose a lifetime risk. Finally, little is known about the harm due to chronic fatigue and neuropsychological sequelae [18]. Figure 3 helps to directly calculate the risk threshold if more conservative assumptions are made. For example, when the relative risk reduction due to HD-CT is only 20% and the harm 8%, only patients with at least a 40% risk will benefit from more aggressive treatment.

Our analysis has some other limitations. To compare harm and benefit of HD-CT we expressed both in 10-year risks, without considering the time of the event since treatment (early or late). This is a simplification. An alternative would be a more extensive decision analysis, in which expected life years and the probability of complications are modelled, e.g. with a Markov model with yearly cycles [33]. However given the uncertainty in the estimates of harm and benefit such a more complicated model was not considered desirable.

We also did not consider costs of HD-CT or SD-CT. There are currently no data available on the difference in costs between HD-CT and SD-CT for testicular cancer patients but in other diseases, such as non-Hodgkin's lymphoma, multiple myeloma and breast cancer, the costs of HD chemotherapy have been reported to be one to four times higher than SD [34]. Hence, HD-CT needs to have a substantial net benefit to be relevant from a societal perspective.

Evidence of the benefit of HD-CT as first line treatment in the literature has not been conclusive, and the results of two ongoing RCTs have to be awaited for more reliable decision making. One RCT by the EORTC (BEP vs. high-dose VIP) is still including poor prognosis patients [8]. The inclusion of intermediate and poor prognosis patients for an RCT by the US intergroup (BEP vs. high-dose CEC) has closed and preliminary results have been presented [9,35]. There was no significant difference in complete response after 1 year between standard and high-dose chemotherapy (48 vs. 52%). We will have to await the publication of the final results of these RCTs before a more precise estimate of the benefit of HD-CT can be made.

Based on the number of patients enrolled in these trials, a relative risk reduction over approximately 50% can be detected with sufficient statistical power. This may be an optimistic estimate, and results of the trials may be inconclusive when HD-CT in fact has a smaller effect. Our analysis suggests that HD-CT may not be beneficial for the full group of intermediate prognosis patients, especially because of excess long-term mortality and morbidity. Special attention should be given to the intermediate prognosis patients in the analysis of the RCT that includes these patients [9]. Further, it is important that more precise information becomes available on the long term complications of HD-CT by longer follow-up, since testicular cancer occurs mostly at a young age.

Besides HD-CT other approaches are being investigated to improve survival of NSGCT patients, such as dose intensification and the introduction of new agents [36-38].

A recently published phase II trial investigating the intensive induction chemotherapy carboplatin, bleomycin, vincristine, cisplatin + bleomycin, etoposide, cisplatin (C-BOP/BEP) showed promising results with 2-year survival of 94 and 85% for intermediate and poor prognosis patients respectively. However, 2-year progression free survival was much lower for poor prognosis patients (56%) suggesting that the benefit will be smaller at 5 or 10-year follow-up [39].

Furthermore the EORTC currently conducts a RCT targeted especially at intermediate prognosis patients which investigates the combination of paclitaxel with BEP (T-BEP) [40].

The results of these trials can be incorporated in the decision analytic approach described in this study to determine which treatment is optimal at what harm and benefit.

## Conclusion

In conclusion, we illustrated how decision analysis can support treatment choices on more aggressive therapy. From the decision analysis we learn at what risk a treatment becomes beneficial. A prognostic model or prognostic classification can then be used to estimate the risk of an individual patient or a subgroup of patients. This approach can be adapted to new results from ongoing trials and extended to many other cancers to explicitly define candidates for more aggressive treatments. Hence, patients who are expected to benefit will be treated more aggressively, without overtreatment of those at relatively low risk, and patients who are not expected to benefit will be treated in a more standard way, without undertreatment of those at relatively high risk.

## Competing interests

The author(s) declare that they have no competing interests.

## Authors' contributions

MvD reviewed the literature on which the estimates of harm and benefit were based and performed the statistical analysis.

All authors have substantially contributed in the design of the study, and in drafting and revising the manuscript.

All authors read and approved the final manuscript.

## Pre-publication history

The pre-publication history for this paper can be accessed here:



## References

[B1] Piccirillo JF, Tierney RM, Costas I, Grove L, Spitznagel EL (2004). Prognostic importance of comorbidity in a hospital-based cancer registry. Jama.

[B2] Simon R, Altman DG (1994). Statistical aspects of prognostic factor studies in oncology. Br J Cancer.

[B3] Harrell FE, Lee KL, Mark DB (1996). Multivariable prognostic models: issues in developing models, evaluating assumptions and adequacy, and measuring and reducing errors. Stat Med.

[B4] Glasziou PP, Irwig LM (1995). An evidence based approach to individualising treatment. Bmj.

[B5] Schmoll HJ, Kollmannsberger C, Metzner B, Hartmann JT, Schleucher N, Schoffski P, Schleicher J, Rick O, Beyer J, Hossfeld D, Kanz L, Berdel WE, Andreesen R, Bokemeyer C (2003). Long-term results of first-line sequential high-dose etoposide, ifosfamide, and cisplatin chemotherapy plus autologous stem cell support for patients with advanced metastatic germ cell cancer: An extended phase I/II study of the German testicular cancer study group. J Clin Oncol.

[B6] Bokemeyer C, Harstrick A, Beyer J, Metzner B, Ruther U, Hartmann JT, Holstein K, Derigs HG, de Wit R, Casper J, Schoffski P, Kuhrer I, Illiger HJ, Kempf B, Reichle A, Foller A, Hossfeld DK, Fischer JT, Berdel W, Gerhartz H, Kirchner H, Pfluger K, Ostermann H, Kanz L, Schmoll HJ (1998). First-line high-dose chemotherapy for 'poor risk' metastatic non-seminomatous testicular germ cell tumors. Onkologie.

[B7] Bokemeyer C, Oechsle K, Hartmann JT, Schoffski P, Schleucher N, Metzner B, Schleicher J, Kanz L (2002). Treatment-induced anaemia and its potential clinical impact in patients receiving sequential high dose chemotherapy for metastatic testicular cancer. Br J Cancer.

[B8] EORTC Phase III Randomized Study of Standard Cisplatin, Etoposide, and Ifosfamide (VIP) Followed By Sequential High-Dose VIP and Stem Cell Rescue Versus Bleomycin, Etoposide, and Cisplatin (BEP) in Chemotherapy-Naive Men With Poor-Prognosis Germ Cell Cancer. http://www.cancer.gov/search/ViewClinicalTrials.aspx?cdrid=67134&version=HealthProfessional&protocolsearchid=1480362.

[B9] USiintergroup (2006). Phase III Randomized Study of Bleomycin, Etoposide, and Cisplatin (BEP) With or Without High-Dose Carboplatin, Etoposide, and Cyclophosphamide Plus Autologous Bone Marrow or Peripheral Blood Stem Cell Transplantation in Male Patients With Previously Untreated Poor- or Intermediate-Risk Germ Cell Tumors.

[B10] Chaudhary UB, Haldas JR (2003). Long-term complications of chemotherapy for germ cell tumours. Drugs.

[B11] IGCCCG (1997). International Germ Cell Consensus Classification: a prognostic factor-based staging system for metastatic germ cell cancers. International Germ Cell Cancer Collaborative Group. J Clin Oncol.

[B12] de Wit R, Roberts JT, Wilkinson PM, de Mulder PH, Mead GM, Fossa SD, Cook P, de Prijck L, Stenning S, Collette L (2001). Equivalence of three or four cycles of bleomycin, etoposide, and cisplatin chemotherapy and of a 3- or 5-day schedule in good-prognosis germ cell cancer: a randomized study of the European Organization for Research and Treatment of Cancer Genitourinary Tract Cancer Cooperative Group and the Medical Research Council. J Clin Oncol.

[B13] Hinton S, Catalano PJ, Einhorn LH, Nichols CR, David Crawford E, Vogelzang N, Trump D, Loehrer PJ (2003). Cisplatin, etoposide and either bleomycin or ifosfamide in the treatment of disseminated germ cell tumors: final analysis of an intergroup trial. Cancer.

[B14] Sonneveld DJ, Hoekstra HJ, Van Der Graaf WT, Sluiter WJ, Schraffordt Koops H, Sleijfer DT (1999). The changing distribution of stage in nonseminomatous testicular germ cell tumours, from 1977 to 1996. BJU Int.

[B15] van Dijk MR, Steyerberg EW, Habbema JD (2006). Survival of non-seminomatous germ cell cancer patients according to the IGCC classification: An update based on meta-analysis. Eur J Cancer.

[B16] Tengs TO, Wallace A (2000). One thousand health-related quality-of-life estimates. Med Care.

[B17] Sonneveld DJA, Hoekstra HJ, van der Graaf WT, Sluiter WJ, Mulder NH, Willemse PHB, Koops HS, Sleijfer DT (2001). Improved long term survival of patients with metastatic nonseminomatous testicular germ cell carcinoma in relation to prognostic classification systems during the cisplatin era. Cancer.

[B18] Bokemeyer C, Kollmannsberger C, Meisner C, Harstrick A, Beyer J, Metzner B, Hartmann JT, Schmoll HJ, Einhorn L, Kanz L, Nichols C (1999). First-line high-dose chemotherapy compared with standard-dose PEB/VIP chemotherapy in patients with advanced germ cell tumors: A multivariate and matched-pair analysis. J Clin Oncol.

[B19] Bosl GJ, Motzer RJ (1997). Testicular germ-cell cancer. N Engl J Med.

[B20] Rosti G, Ferrante P, Ledermann J, Leyvraz S, Ladenstein R, Koscileniak E, Crown J, Dazzi C, Cariello A, Marangolo M (2002). High-dose chemotherapy for solid tumors: results of the EBMT. Crit Rev Oncol Hematol.

[B21] Bokemeyer C, Berger CC, Kuczyk MA, Schmoll HJ (1996). Evaluation of long-term toxicity after chemotherapy for testicular cancer. J Clin Oncol.

[B22] Kollmannsberger C, Hartmann JT, Kanz L, Bokemeyer C (1999). Therapy-related malignancies following treatment of germ cell cancer. Int J Cancer.

[B23] Xie Y, Davies SM, Xiang Y, Robison LL, Ross JA (2003). Trends in leukemia incidence and survival in the United States (1973-1998). Cancer.

[B24] Huddart RA, Norman A, Shahidi M, Horwich A, Coward D, Nicholls J, Dearnaley DP (2003). Cardiovascular disease as a long-term complication of treatment for testicular cancer. J Clin Oncol.

[B25] Meinardi MT, Gietema JA, van der Graaf WT, van Veldhuisen DJ, Runne MA, Sluiter WJ, de Vries EG, Willemse PB, Mulder NH, van den Berg MP, Koops HS, Sleijfer DT (2000). Cardiovascular morbidity in long-term survivors of metastatic testicular cancer. J Clin Oncol.

[B26] Jonker-Pool G, Van de Wiel HB, Hoekstra HJ, Sleijfer DT, Van Driel MF, Van Basten JP, Schraffordt Koops HS (2001). Sexual functioning after treatment for testicular cancer--review and meta-analysis of 36 empirical studies between 1975-2000. Arch Sex Behav.

[B27] Redaelli A, Stephens JM, Brandt S, Botteman MF, Pashos CL (2004). Short- and long-term effects of acute myeloid leukemia on patient health-related quality of life. Cancer Treat Rev.

[B28] Vickers AJ, Kramer BS, Baker SG (2006). Selecting patients for randomized trials: a systematic approach based on risk group. Trials.

[B29] van Dijk MR, Steyerberg EW, Stenning SP, Dusseldorp E, Habbema JD (2004). Survival of patients with nonseminomatous germ cell cancer: a review of the IGCC classification by Cox regression and recursive partitioning. Br J Cancer.

[B30] van Dijk MR, Steyerberg EW, Stenning SP, Habbema JD (2006). Survival estimates of a prognostic classification depended more on year of treatment than on imputation of missing values. J Clin Epidemiol.

[B31] Donadio AC, Bosl GJ (2002). The future of therapy for nonseminomatous germ cell tumors. Chest Surg Clin N Am.

[B32] Heidenreich A, Srivastava S, Moul JW, Hofmann R (2000). Molecular genetic parameters in pathogenesis and prognosis of testicular germ cell tumors. Eur Urol.

[B33] Sonnenberg FA, Beck JR (1993). Markov models in medical decision making: a practical guide. Med Decis Making.

[B34] Simnett SJ, Stewart LA, Sweetenham J, Morgan G, Johnson PW (2000). Autologous stem cell transplantation for malignancy: a systematic review of the literature. Clin Lab Haematol.

[B35] Bajorin DF, Nichols CR, Margolin KA, Bacik J, Richardson PG, Vogelzang NJ, Einhorn L, Mazumdar M, Bosl GJ, Motzer RJ Phase III trial of conventional-dose chemotherapy alone or with high-dose chemotherapy for metastatic germ cell tumors (GCT) patients (PTS): A cooperative group trial by Memorial Sloan-Kettering Cancer Center, ECOG, SWOG, and CALGB. J Clin Oncol.

[B36] Bower M, Newlands ES, Holden L, Rustin GJ, Begent RH (1997). Treatment of men with metastatic non-seminomatous germ cell tumours with cyclical POMB/ACE chemotherapy. Ann Oncol.

[B37] Germa-Lluch JR, Garcia del Muro X, Tabernero JM, Sanchez M, Aparicio J, Alba E, Barnadas A (1999). BOMP/EPI intensive alternating chemotherapy for IGCCC poor-prognosis germ-cell tumors: the Spanish Germ-Cell Cancer Group experience (GG). Ann Oncol.

[B38] Kaye SB, Mead GM, Fossa S, Cullen M, deWit R, Bodrogi I, van Groeningen C, Sylvester R, Collette L, Stenning S, De Prijck L, Lallemand E, deMulder P (1998). Intensive induction-sequential chemotherapy with BOP/VIP-B compared with treatment with BEP/EP for poor-prognosis metastatic nonseminomatous germ cell tumor: a Randomized Medical Research Council/European Organization for Research and Treatment of Cancer study. J Clin Oncol.

[B39] Fosså SD, Paluchowska B, Horwich A, Kaiser G, de Mulder PH, Koriakine O, van Oosterom AT, de Prijck L, Collette L, de Wit R, EORTC GU Group (2005). Intensive induction chemotherapy with C-BOP/BEP for intermediate- and poor-risk metastatic germ cell tumours (EORTC trial 30948). Br J Cancer.

[B40] EORTC Phase II/III Randomized Study of Bleomycin, Cisplatin, and Etoposide (BEP) Versus Bleomycin, Cisplatin, Etoposide, and Paclitaxel (T-BEP) in Men With Intermediate Prognosis Germ Cell Cancer. http://www.cancer.gov/search/ViewClinicalTrials.aspx?cdrid=66731&protocolsearchid=1937772&version=healthprofessional.

[B41] Hartmann JT, Albrecht C, Schmoll HJ, Kuczyk MA, Kollmannsberger C, Bokemeyer C (1999). Long-term effects on sexual function and fertility after treatment of testicular cancer. Br J Cancer.

